# Effects of Mephedrone and Amphetamine Exposure during Adolescence on Spatial Memory in Adulthood: Behavioral and Neurochemical Analysis

**DOI:** 10.3390/ijms22020589

**Published:** 2021-01-08

**Authors:** Pawel Grochecki, Irena Smaga, Malgorzata Lopatynska-Mazurek, Ewa Gibula-Tarlowska, Ewa Kedzierska, Joanna Listos, Sylwia Talarek, Marta Marszalek-Grabska, Magdalena Hubalewska-Mazgaj, Agnieszka Korga-Plewko, Jaroslaw Dudka, Zbigniew Marzec, Małgorzata Filip, Jolanta H. Kotlinska

**Affiliations:** 1 Department of Pharmacology and Pharmacodynamics, Medical University, 20-093 Lublin, Poland; pa.grochecki@gmail.com (P.G.); gosia.lopatynska@gmail.com (M.L.-M.); ewa.gibula@umlub.pl (E.G.-T.); ewa.kedzierska@umlub.pl (E.K.); joanna.listos@umlub.pl (J.L.); sylwia.talarek@umlub.pl (S.T.); 2 Department of Drug Addiction Pharmacology, Polish Academy of Sciences, 31-343 Krakow, Poland; smaga@if-pan.krakow.pl (I.S.); hubalew@if-pan.krakow.pl (M.H.-M.); mal.fil@if-pan.krakow.pl (M.F.); 3 Department of Experimental and Clinical Pharmacology, Medical University, 20-090 Lublin, Poland; martamarszalekgrabska@umlub.pl; 4 Independent Medical Biology Unit, Medical University, 20-090 Lublin, Poland; agnieszka.korga-plewko@umlub.pl; 5 Department of Toxicology, Medical University, 20-090 Lublin, Poland; jaroslaw.dudka@umlub.pl; 6 Department of Food and Nutrition, Medical University, 20-093 Lublin, Poland; zbigniew.marzec@umlub.pl

**Keywords:** mephedrone, spatial memory, MMP-9, NMDA, young adult rats

## Abstract

A synthetic cathinone, mephedrone is widely abused by adolescents and young adults. Despite its widespread use, little is known regarding its long-term effects on cognitive function. Therefore, we assessed, for the first time, whether (A) repeated mephedrone (30 mg/kg, i.p., 10 days, once a day) exposure during adolescence (PND 40) induces deleterious effects on spatial memory and reversal learning (Barnes maze task) in adult (PND 71–84) rats and whether (B) these effects were comparable to amphetamine (2.5 mg/kg, i.p.). Furthermore, the influence of these drugs on MMP-9, NMDA receptor subunits (GluN1, GluN2A/2B) and PSD-95 protein expression were assessed in adult rats. The drug effects were evaluated at doses that per se induce rewarding/reinforcing effects in rats. Our results showed deficits in spatial memory (delayed effect of amphetamine) and reversal learning in adult rats that received mephedrone/amphetamine in adolescence. However, the reversal learning impairment may actually have been due to spatial learning rather than cognitive flexibility impairments. Furthermore, mephedrone, but not amphetamine, enhanced with delayed onset, MMP-9 levels in the prefrontal cortex and the hippocampus. Mephedrone given during adolescence induced changes in MMP-9 level and up-regulation of the GluN2B-containing NMDA receptor (prefrontal cortex and hippocampus) in young adult (PND 63) and adult (PND 87) rats. Finally, in adult rats, PSD-95 expression was increased in the prefrontal cortex and decreased in the hippocampus. In contrast, in adult rats exposed to amphetamine in adolescence, GluN2A subunit and PSD-95 expression were decreased (down-regulated) in the hippocampus. Thus, in mephedrone—but not amphetamine-treated rats, the deleterious effects on spatial memory were associated with changes in MMP-9 level. Because the GluN2B-containing NMDA receptor dominates in adolescence, mephedrone seems to induce more harmful effects on cognition than amphetamine does during this period of life.

## 1. Introduction

During adolescence, the brain continues to undergo important maturation processes (e.g., a decrease in gray matter and an increase in white matter) [1,2,3] that are linked with striking changes in behavior and cognitive function [4,5]. Unfortunately, neurobiological development during adolescence can be influenced by environmental factors such as legal (alcohol and nicotine) and illegal (e.g., cocaine and methamphetamines) drug exposure. Research suggests that drug abuse in adolescence increases risk of illicit drug use in adulthood [6]. Current findings also suggest that learning and memory are vulnerable to adolescent drug use, and deficits in learning and memory following adolescent drug use endure into adulthood [7,8,9,10].

The neuroanatomical contributors of learning and memory are abundant, as cognition is a multisystem behavior. Herein, the prefrontal cortex (PC), and hippocampus (HC) predominate. Animal studies have demonstrated that various sub-regions within the PC play a critical role in behavioral flexibility and attention [11,12,13,14], whereas sub-regions of the HP appear to be vital in spatial and contextual learning and memory [15,16]. In adolescent and adult rodents, the acute administration of an abused drug alters aspects of learning and memory associated with PC and HC [17,18,19,20,21]. Due to the continuous development of these regions during the adolescent time period, these acute alterations may disturb typical development and ultimately manifest as persistent cognitive deficits.

Mephedrone (4-methylmethcathinone) is a synthetic stimulant drug belonging to the group of beta-cathinone derivatives. The chemical structure of mephedrone is closely related to the phenylethylamine family of illegal drugs, including methamphetamine and 3,4-methylenedioxy methamphetamine (MDMA) [22]. Pharmacologically, mephedrone acts as a non-selective, amphetamine-like substrate at monoamine transporters, thereby evoking the release of serotonin, dopamine and (likely) norepinephrine [23,24,25,26]. Recently, abuse of synthetic cathinone, in particular, mephedrone, has increased among adolescents and young adults [27,28]. In controlled oral administration, mephedrone was found to induce euphoric effects, elevated mood and stimulation with a high abuse liability characterized by earlier onset and shorter duration (t_1/2_ = 2.15 h [29]), in comparison to 3,4-methylenedioxymethamphetamine (MDMA) (t_1/2_ = 7.88 h [29]) and other amphetamine derivatives [30,31,32]. Therefore, mephedrone users experience a strong desire to “redose”, leading them to ingest large amounts of the drug in binges that can last several days [33].

Mephedrone use in humans is associated with deficits in working memory and verbal recall [34,35,36,37]. In adolescent [38,39,40] and adult [37] rodents, mephedrone exposure induces deficits in working memory, object recognition and discrimination and selectively impairs retention of fear motivated contextual memory [41,42]. These mephedrone-induced cognitive deficits can be often accompanied by changes in striatal levels of proteins involved in monoamine transmission, such as presynaptic dopamine or serotonin transporters (DAT or SERT, respectively, as reviewed in Angoa-Perez et al. [43]). However, preclinical studies have also shown that mephedrone treatment can cause long-term memory impairment that is not associated with residual changes in monoamine tissue concentrations [37,38].

Matrix metalloproteinases (MMPs) are a family of proteases that degrade components of the extracellular matrix (ECM) to allow for central nervous system (CNS) reorganization. The MMPs subfamily of gelatinases, the most prominent being MMP-2 and MMP-9, are to date the most studied. MMP-9 (gelatinase B) is a zinc-dependent endopeptidase found in the hippocampus, cerebellum, and cerebral cortex [44,45,46,47] and is predominantly expressed by neurons, but also by glia [45,48]. During early postnatal periods (called “critical periods”), MMP-9 is crucial for brain development due to its association with important neurophysiological functions, such as synaptic plasticity [49,50] and long-term potentiation [51,52]. In the adult brain, constitutive levels of this proteinase are low, however, MMP-9 expression is particularly increased [45,53] and further enhanced following learning [54] or synaptic potentiation [55]. Published data demonstrated that MMP-9 has emerged as a physiological regulator of N-methyl-D-aspartate (NMDA)-dependent synaptic plasticity [53]. In contrast, dysregulation of MMP-9 levels (with overactivity in same conditions) leads to a variety of pathologies such as schizophrenia, epilepsy, fragile X syndrome, Alzheimer’s disease, and depression [56,57,58]. Abnormal MMP-9 activity can also contribute to cognitive dysfunction [49]. It has been indicated that both total genetic deletion of MMP-9 (mice) and a 15-fold overexpression of MMP-9 (rats) impair the maintenance of LTP in the mossy fiber-CA3 pathway in hippocampal adolescent slices [59]. Of note, published data indicated that substance abuse [60], including mephedrone [42], modifies MMP-9 level in different brain structures. However, the influence of chronic mephedrone administration during adolescence on MMP-9 level and memory functioning in adults has not yet been evaluated.

The Barnes maze is one of the main behavioral tasks use to study spatial learning and memory in rodents [61]. This maze is a dryland-based behavioral paradigm for assessing spatial learning and memory in rodent. It represents a well-established alternative to the more popular Morris Water maze and offers the advantage of being free from the potentially confounding influence of swimming behavior [62]. In current study, this task was used to assess two hypotheses, whether (1) mephedrone given repeatedly during adolescence induces memory impairment in adult rats, and whether (2) the effects of mephedrone are similar/comparable to those of amphetamine. In addition, we performed a set of neurochemical experiments to examine the effects of mephedrone and amphetamine on the level of MMP-9 (with ELISA test) and N-methyl-D-aspartate (NMDA) glutamate receptor subunits (GluN1, GluN2A, and GluN2B), and the expression of postsynaptic density protein-95 (PSD-95) (Western blot assay) in the PC and HC in adult rats.

## 2. Materials and Methods

### 2.1. Animals

Male Wistar rats (135–170 g; postnatal day—PND 40—at the beginning of the experiments [63]) (OMD, Lublin, Poland) were used, as at this age, animals exhibit behavioral, neurochemical, and endocrine patterns similar to those seen in human adolescent subjects [64]. The 96 animals were kept (2 or 3 individuals) in cages (55 cm × 33 cm × 20 cm) under standard laboratory conditions: a constant temperature of 22 ± 1 °C, controlled humidity within 55% ± 10%, natural day–night cycle (12 h/12 h) and free access to drinking water and standard laboratory chow (Sniff Spezialdiäten GmbH, Soest, Germany). The rats were handled once a day for five days before the beginning of the behavioral experiments. After this procedure, the rats were divided randomly into two groups: behavioral experiments (*n* = 24) and biochemical experiments (*n* = 72). All studies were performed between 08:00 a.m. and 7:00 p.m., were approved by the Local Ethic Committee and were carried out according to the National Institute of Health Guidelines for the Care and Use of Laboratory Animals and the European Community Council Directive of November 2010.

### 2.2. Drugs

Mephedrone hydrochloride (Tocris Bioscience, Bristol, UK) and amphetamine hydrochloride (Tocris Bioscience, Bristol, UK) were dissolved in sterile 0.9% saline (0.9% NaCl, Baxter, Warsaw, Poland). Saline solutions of mephedrone and amphetamine were prepared ex tempore before each administration. All rats were treated intraperitoneally (i.p.), with mephedrone (30 mg/kg) and amphetamine (2.5 mg/kg), once a day for 10 consecutive days, beginning from PND 40. The multiple dose/day mephedrone administration schedule was based on Motbey et al. [63]. All drug doses were chosen based on previous literature data that confirm the rewarding effect of these drugs in the CPP paradigm in rats [65,66]. The dose of mephedrone may be considered to represent typical human recreational mephedrone use [40].

### 2.3. Procedures

#### 2.3.1. Barnes Maze Task

The Barnes maze task was carried out according to the method described by Gawel et al. [61]. The Barnes maze apparatus (Stoelting, Dublin, Ireland) is a round platform made of gray metal (122 cm in diameter) placed 90 cm above the floor. On the surface of the platform there are 20 holes arranged at its edges at equal intervals. Only one hole is connected to an escape box (35 × 12 × 12 cm) made of the same material and color as the platform. The remaining holes are covered. Visual cues are placed on the walls of the experimental room, at 1–2 m distance from the edge of the maze (large, colorful geometrical figures). One potential drawback of the Barnes maze is that the lack of stressful stimuli can result in slow learning. In order to provoke potentiated escape response into the escape box, the platform is brightly lit (two light points 1.5 m above the platform, 500 W each) and a buzzer placed above the center provides a sound of 80 dB. The buzzer provides mild stress and increases motivation for escape during all trials. Other groups have used buzzer noise in a similar manner to induce escape behavior [67,68].

In our study, the Barnes maze task was run in the following phases: habituation, acquisition, probe trial 1 (24 h after acquisition), probe trial 2 (14 days after acquisition), and reversal learning (24 h after the probe trial 2). In each of the phases of the task, excluding habituation, the time to find the entrance to the escape box (primary latency) and the number of primary errors made at that time were measured. All trials were recorded and analyzed using video-tracking software (Karnet, Lublin, Poland).

#### 2.3.2. Habituation

The habituation phase was conducted one day prior to the acquisition phase. This phase was introduced to minimize animal anxiety behavior and to acquaint the animal with the apparatus. Each animal was placed in the center of the platform and left for 180 s to explore freely (with buzzer switched off). After this time, the rat was placed back in its home cage, and the platform surface was rinsed with a solution of ethanol to remove dirt and odorants. Habituation was carried out with the light on, but the buzzer was turned off.

#### 2.3.3. Acquisition phase

This part of the experiment began 24 h after the habituation phase. This step enabled the rats to learn to locate the position of the escape box. There were two acquisition-training sessions (180 s each trial) per day for 4 consecutive days with a 5 min inter-trial interval during which the animals were returned to their home cage. The location of the platform and the escape box remained constant over the complete acquisition trial. Each trial started by placing the animal at the center of the platform, with the buzzer switched on and the rats allowed to freely explore the apparatus. The trial was terminated after 180 s or when the animal entered the escape box. The buzzer was then switched off and the hole was covered for 30 s before the rat was returned its home cage. However, when an animal did not enter the escape box within 180 s, the experimenter gently guided it to there. To minimize odor cues and provide a standard olfactory context for each trial, the apparatus surface and escape box were wiped with 10% (*w*/*v*) ethanol solution after every trial. After the acquisition phase, spatial memory was evaluated.

#### 2.3.4. Probe Trial

The probe trial was performed 24 h (Probe 1) and 14 days (Probe 2) after the acquisition training. Here, the escape box location remained the same as during the training sessions, however, for the probe trial, access to the escape box was blocked. This test measures the rat spatial memory retrieval (memory retention). Each session lasted 90 s. The experiment was carried out with the light and buzzer switched on. Primary latency and primary errors to reach the escape box were evaluated.

#### 2.3.5. Reversal Learning

The goal of this phase was to assess memory flexibility. This phenomenon can be defined as the ability to change the learned behavioral schedule. Reversal learning was carried out 1 day after completing the second probe trial. Two sessions per day were performed for each animal for three consecutive days. The experimental conditions were the same as during the acquisition phase (with the buzzer switched on), except that the position of the escape box was rotated 180 degrees. The rat was, therefore, unable to find the escape box using the acquired spatial cues but had to relearn the new location of the hole. In the reversal-learning phase of the task, primary latency, and primary errors to reach the escape box were counted.

### 2.4. Locomotor Activity Test

The rats were placed individually in a locomotor apparatus (Porfex, Białystok, Poland), which is a square cage, 60 cm a side, made of transparent plastic. The horizontal activity of the animals was measured using infrared sensors placed 45 and 100 mm above the floor. The locomotor activity of each rat, expressed as the distance travelled in meters (m), was recorded for 15 min. The test was carried out in a soundproof experimental room lit by a dimmed (red bulb) light. After each session, the apparatus was thoroughly cleaned with ethanol solution to remove olfactory stimuli.

### 2.5. Biochemical Experiments

#### 2.5.1. Western Blot

Frozen brain structures were homogenized in cold 0.32 M sucrose buffer pH 7.4 containing 1 mM HEPES, 1 mM MgCl_2_, 1 mM NaHCO_3_, and 0.1 mM PMSF, in the presence of cocktails of protease and phosphatase (Sigma-Aldrich) inhibitors, using a homogenizer ball (Bioprep-24, Allsheng, China) (10 s at 10,000 rpm). For protein determination, a bicinchoninic acid assay (BCA) protein assay kit (Serva, Heidelberg, Germany) was used. Homogenate (10 μg of protein) was then denatured and resolved by 10% SDS polyacrylamide gels and transferred to a polyvinylidene difluoride (PVDF) membrane. Membranes were blocked in 3% non-fat dry milk, and separate sets of membranes were probed with mouse anti-GluNR1 monoclonal antibody (1:1000; 32-0500, Thermo Fisher ScienPSDic, Waltham, MA, USA), rabbit anti-GluNR2A polyclonal antibody (A-6473; 1:1000; Thermo Fisher ScienPSDic, Waltham, MA, USA), rabbit anti-GluNR2B polyclonal antibody (1:1000; ab65783; Abcam, Cambridge, UK), and rabbit anti-PSD-95 polyclonal antibody (1:4000; #3450; Cell Signaling Technology, Denvers, CO, USA). The expressions of NMDA receptor subunits and scaffolding protein were evaluated relative to that of β-actin control protein, using mouse monoclonal antibody at dilution of 1:1000 (A5441; Sigma-Aldrich, Saint Louis, MO, USA). Blots were washed and incubated with donkey goat anti-rabbit secondary antibody (1:6000; 926-68071; Li-Cor, Lincoln, NE, USA) or goat anti-mouse (1:6000; 926-32210; Li-Cor, Lincoln, NE, USA) and visualized using fluorescence detection Odyssey Clx (Li-Cor, Lincoln, NE, USA). Analysis was performed by Image Studio v.2.1. All data were expressed as % of control.

#### 2.5.2. ELISA Assay

Quantitative measurement of MMP-9 in tissue homogenates was performed using a Rat Matrix Metalloproteinase 9 (MMP-9) ELISA Kit (Reddot Biotech, Kelowna, BC, Canada), following manufacturer’s protocol. Firstly, homogenates (see Section 2.5.1. Western blot) were centrifuged for 5 min at 5000× *g*. After this, the supernates were immediately removed and protein concentration was determined in each sample with a bicinchoninic acid (BCA) protein assay kit (Serva, Heidelberg, Germany). From each sample, 100 μg of protein was used in the ELISA assay. All data were expressed in ng/mL.

#### 2.5.3. Experimental Design

##### Experiment 1

At PND 40, rats (*n* = 8/group) were treated with saline, mephedrone (30 mg/kg, i.p.) and amphetamine (2.5 mg/kg, i.p.), once a day for 10 consecutive days. Fifteen days after the last injection, they were subjected to Barnes maze task (PND 66), and the habituation (1 day, PND 66), acquisition (4 days, PND 67,68,69,70), probe trial 1 (1 day, PND 71), probe trial 2 (1 day, PND 84) and reversal learning (3 days, PND 85–87) were conducted. Locomotor activity was assessed on PND 71, immediately after probe trial 1.

After completion of the Barnes maze procedure, all animals (PND 87) were killed by decapitation, and brain tissue (PC and HC) were collected for neurochemical assessment (MMP-9, PSD-95, and NMDA receptor subunits proteins—GluN1, GluN2A, GluN2B).

##### Experiment 2

**Figure 1:**
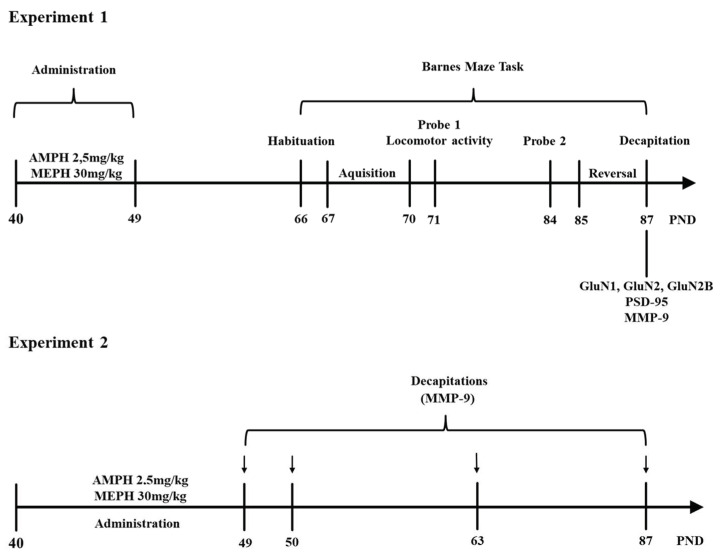
Diagram of experimental designs 1 and 2. AMPH—amphetamine; MEPH—mephedrone; PND—postnatal day; MMP-9—matrix metalloproteinase-9.

In these studies, the following animal groups (*n* = 6/group) were used: saline (control), mephedrone- and amphetamine-treated. These animals (PND 40) received the saline/drug injections for 10 consecutive days, once daily. They were then sacrificed 2 h, 24 h, 14 days, and 38 days after the last injection. All animals were decapitated, and their brains were quickly removed and chilled in ice-cold saline. The PC and HC were dissected. Samples were immediately frozen in liquid nitrogen and stored at −80 °C for later analysis of MMP-9 expression (with ELISA test) and GluN2B subunit (Western blot assay) of NMDA receptor (PND 63) (see Figure 1).

### 2.6. Statistical Analysis

Statistical analysis of results was performed using the one- and two-factor analysis of variance (one-way ANOVA and two-way ANOVA) or Student’s *t*-test). Comparisons between groups were made by applying the Bonferroni or Dunnett’s post-hoc test. Differences were considered statistically significant when the determined *p*-value was less than 0.05 (*p* < 0.05). All data were analyzed using Prism v. 8.0.0 for Windows (GraphPad Software, San Diego, CA, USA) and presented as mean ± SEM.

## 3. Results

### 3.1. Experiment 1

#### 3.1.1. The Influence of Repeated Mephedrone and Amphetamine Administration during Late Adolescence on Acquisition Memory of the Barnes Maze Task in Adult Rats

**Figure 2:**
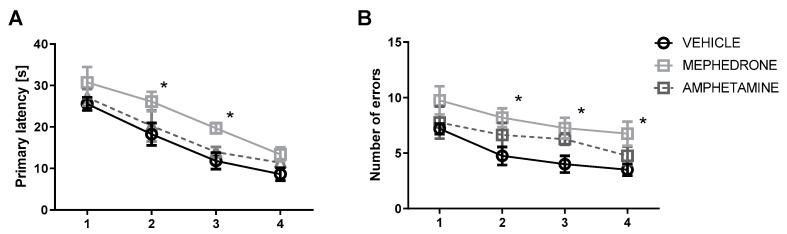
Effect of repeated mephedrone or amphetamine administration on (**A**) primary latency and (**B**) number of errors committed during 4 days of acquisition training of the Barnes maze task (PND 67-70). Data are expressed as the mean ± SEM. *n* = 8 rats/group. * *p* < 0.05 vs. vehicle (0.9% NaCl).

Acquisition of spatial memory in the training phase was evaluated by the decrease in the number of errors and latency time to reach the escape box for four days (PND 67–70). In the primary latency, a two-way ANOVA with repeated measures showed significant effects of group (F (2.84) = 8.401, *p* < 0.001) and day of acquisition learning (F (3.84) = 31.55, *p* < 0.0001) but no group × day of interaction (F (6.84) = 0.09108, *p* > 0.05; *n* = 8/group). In addition, in the number of primary errors committed, a two-way ANOVA with repeated measures indicated significant effect of group (F (2.84) = 11.32; *p* < 0.0001) and day of acquisition learning (F (3.84) = 6.533, *p* < 0.001), but no group x day of interaction (F (6.84) = 0.1778, *p* > 0.05; *n* = 8/group). Bonferroni post-hoc test showed that repeated mephedrone but not amphetamine administration impaired acquisition of spatial learning. This effect was observed as the increases in primary latency in mephedrone-treated rats in the second and third (*p* < 0.05) day of acquisition learning (Figure 2A). In the mephedrone-treated rats, Bonferroni post-hoc test revealed a significant increase in the number of errors committed on the 2nd (*p* < 0.05), 3rd (*p* < 0.05) and 4th day (*p* < 0.05) of spatial learning in reaching the target hole (Figure 2B).

#### 3.1.2. The Influence of Repeated Mephedrone and Amphetamine Administration during Late Adolescence on Spatial Memory Retrieval in Probe Trial 1 and Probe Trial 2 of the Barnes Maze Task in Adult Rats

**Figure 3:**
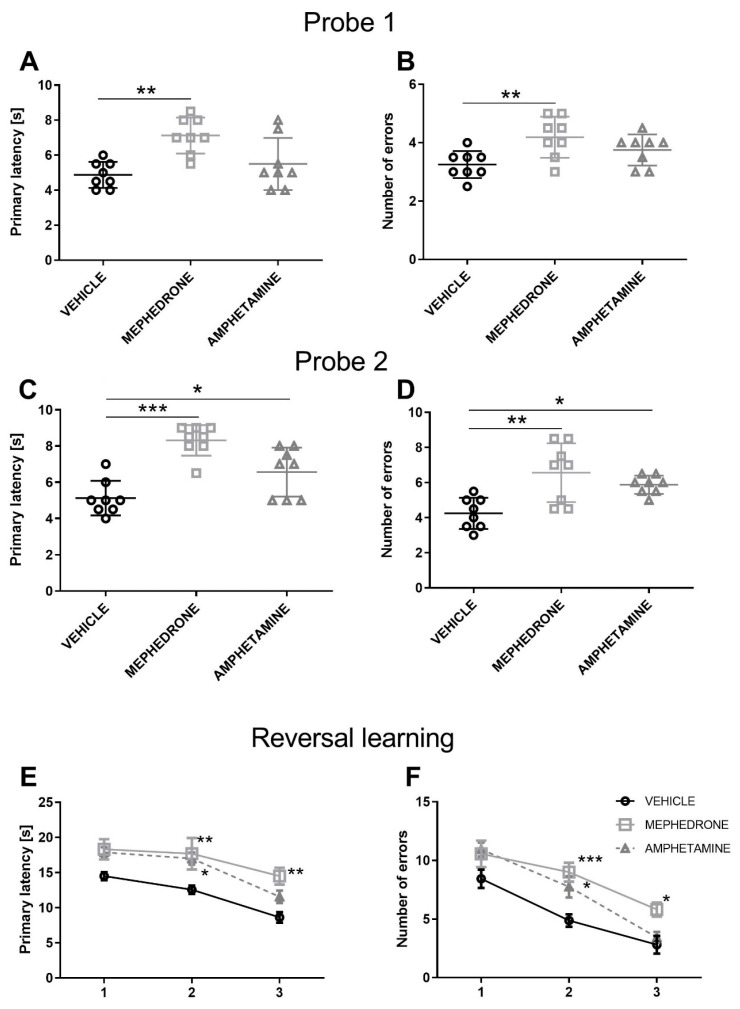
Effect of repeated mephedrone or amphetamine administration on (**A**) primary latency and (**B**) number of errors committed in the probe trial (Probe 1) of the Barnes maze task (PND 71); (**C**) primary latency and (**D**) number of errors committed in the probe trial (Probe 2) (PND 84); (**E**) primary latency and (**F**) number of errors committed during 3 days of the reversal learning phase of the Barnes maze task (PND 85–87). Data are expressed as the mean ± SEM. *n* = 8 rats/group. * *p* < 0.05, ** *p* < 0.01, *** *p* < 0.001 vs. vehicle (0.9% NaCl).

One day after completion of acquisition, probe trial 1 (PND 71) was performed to assess spatial memory (memory retention). A one-way ANOVA showed statistically significant differences in primary latency (F (2.21) = 8.472, *p* < 0.01) (Figure 3A) and significant differences in the number of committed errors (F (2.21) = 5.305, *p* < 0.05) (Figure 3B). Bonferroni post-hoc test showed a statistically significant increase in the primary latency and number of committed errors of animals receiving mephedrone (*p* < 0.01), but not amphetamine (*p* > 0.05) during adolescence—as compared to the control group (Figure 3A,B).

Probe trial 2 was performed 14 days after completing the acquisition phase (PND 84) to assess spatial reference memory (long-term memory retention). A one-way ANOVA showed significant differences in the primary latency (F (2.21) = 17.79, *p* < 0.001) and the number of errors (F (2.21) = 8.774, *p* < 0.01). Bonferroni post-hoc test showed that repeated mephedrone/amphetamine administration during adolescence impaired the spatial reference memory in the Barnes maze task. This effect was observed as a significant increase in the primary latency in both mephedrone-(*p* < 0.001) and amphetamine-treated (*p* < 0.05) groups (Figure 3C). In addition, in probe trial 2, rats receiving repeated mephedrone (*p* < 0.01) and amphetamine (*p* > 0.05) administration during adolescence committed more errors than their control counterparts (Figure 3D).

#### 3.1.3. The Influence of Repeated Mephedrone and Amphetamine Administration during Late Adolescence on Reversal Learning of the Barnes Maze Task in Adult Rats

Reversal learning trials were performed one day after probe trial 2 (PND 85–87). In the primary latency, a two-way ANOVA with repeated measures showed significant effects of group (F (2.63) = 14.86, *p* < 0.001) and day of reversal learning (F (2.63) = 18.00, *p* < 0.001), but no group × day of reversal learning interaction (F (4.63) = 0.4543, *p* > 0.05; *n* = 8/group) (Figure 3E). In addition, in the number of primary errors committed, a two-way ANOVA with repeated measures indicated significant effect of group (F (2.63) = 12.81; *p* < 0.001) and day of reversal learning (F (2.63) = 46.56, *p* < 0.001), but no group x day of reversal learning interaction (F (4.63) = 1.271, *p* > 0.05; *n* = 8/group). Bonferroni post-hoc test showed that repeated mephedrone/amphetamine administration impaired reversal learning. This effect was observed as the increases in primary latency in mephedrone-treated rats in the second and third (*p* < 0.01) day of reversal learning. This effect was also observed in the amphetamine-treated rats in the 2nd day of reversal learning (*p* < 0.05) (Figure 3E). In the mephedrone-treated rats, Bonferroni post-hoc test revealed a significant increase in the number of errors committed on the second (*p* < 0.001) and third day (*p* < 0.05) of reversal trials in reaching the target hole. The increase in the number of errors was observed only on the second day of reversal learning (*p* < 0.05) trials in the amphetamine-treated rats (Figure 3F).

#### 3.1.4. The Influence of Repeated Mephedrone and Amphetamine Administration during Late Adolescence on the Expression of NMDA Receptor Subunits (GluN1, GluN2A, and GluN2B), PSD-95, and MMP-9 Proteins in the PC and HC of Adult Rats That Underwent the Barnes-Maze Task

**Figure 4:**
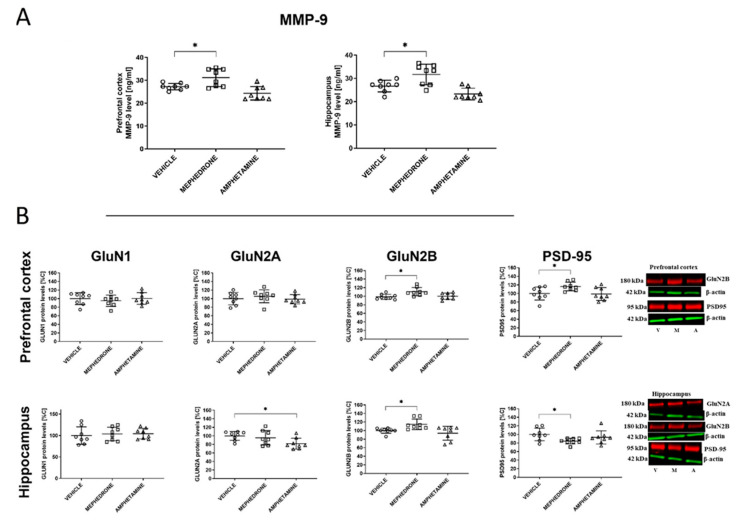
Effect of repeated mephedrone or amphetamine administration during adolescence on MMP-9 (**A**) and NMDA subunits (GluN1, GluN2A, GluN2B) and PSD-95 (**B**) expression in the prefrontal cortex (PC) and hippocampus (HC) of rats (PND 87) that underwent the Barnes maze task. Representative blots for significant changes are presented, as well as full membranes are presented in the supplementary material (Figure S1). Data are expressed as mean ± SEM. *n* = 8 rats/group. * *p* < 0.05 vs vehicle (0.9% NaCl). V—vehicle, M—mephedrone, A—amphetamine.

Mephedrone (10 × 30 mg/kg, i.p.) or amphetamine (10 × 2.5 mg/kg, i.p.) given repeatedly to adolescent rats had impact on the expression of MMP-9 in the prefrontal cortex. One-way ANOVA revealed significant changes after 38 days (F(2,21) = 11.09; *p* < 0.001) of mephedrone withdrawal. Beyond this, Dunnett’s post-hoc test revealed that mephedrone, but not amphetamine, increased MMP-9 level (*p* < 0.05) 38 days after the last administration in the PC (Figure 4A).

One-way ANOVA did not indicate a significant effect in the mephedrone/amphetamine administration on the GluN1 (F(2,21) = 0.3834; *p* > 0.05; *n* = 8) and GluN2A (F(2,21) = 0.5685; *p* > 0.05; *n* = 8) subunits of the NMDA receptor. However, one-way ANOVA indicated significant changes in the GluN2B (F(2,21) = 5.648; *p* < 0.05; *n* = 8) and PSD-95 (F(2,21) = 4.103; *p* < 0.05; *n* = 8) protein expression. In addition, Dunnett’s post-hoc test indicated that mephedrone increased the GluN2B subunit of the NMDA receptor expression and the PSD-95 expression (*p* < 0.05) (Figure 4B).

Mephedrone (10 × 30 mg/kg, i.p.) but not amphetamine (10 × 2.5 mg/kg, i.p.) given repeatedly to adolescent rats had impact on the expression of MMP-9 in the HC. Here, one-way ANOVA revealed significant changes after 38 days (F(2,21) = 13.09; *p* < 0.001) of withdrawal. Moreover, Dunnett’s post-hoc test revealed that mephedrone, but not amphetamine, increased MMP-9 level (*p* < 0.05) in the HC 38 days after the last administration (Figure 4A).

One-way ANOVA did not indicate a significant effect in the administration of mephedrone/amphetamine on the GluN1 (F(2,21) = 0.1644; *p* > 0.05; *n* = 8) subunits of the NMDA receptor and on PSD-95 (F(2,21) = 2.937; *p* > 0.05; *n* = 8) expression. However, one-way ANOVA indicated significant changes in GluN2A (F(2,21) = 3.783; *p* > 0.05; *n* = 8) and GluN2B (F(2,21) = 6.162; *p* < 0.01; *n* = 8) protein expression. Dunnett’s post-hoc test also indicated that mephedrone increased the protein level for the GluN2B subunit of the NMDA receptor (*p* < 0.05) and decreased PSD-95 expression. Furthermore, Dunnett’s post-hoc test indicated that amphetamine decreased the protein level of the GluN2A subunit of the NMDA receptor (*p* < 0.05) (Figure 4B).

#### 3.1.5. The Influence of Repeated Mephedrone and Amphetamine Exposure during Late Adolescence on Adult Rat Locomotor Activity

One-way ANOVA (F (2,21) = 0.3441, *p* > 0.05) and Bonferroni post-hoc test (*p* > 0.05) did not show statistically significant differences in the distance travelled by the adult animals that received mephedrone/amphetamine repeatedly during adolescence—when compared to the control group (Table 1). The locomotor activity test was performed after probe trial 1 (PND 71) of the Barnes maze task.

### 3.2. Experiment 2

The Influence of Repeated Mephedrone and Amphetamine Exposure During Late Adolescence on the MMP-9 Expression in Rat Brain (PC and HC) at 2 h, 24 h, 14, and 38 Days after the Last Drug Administration in Rats that did not Undergo the Barnes Maze Task.

**Figure 5:**
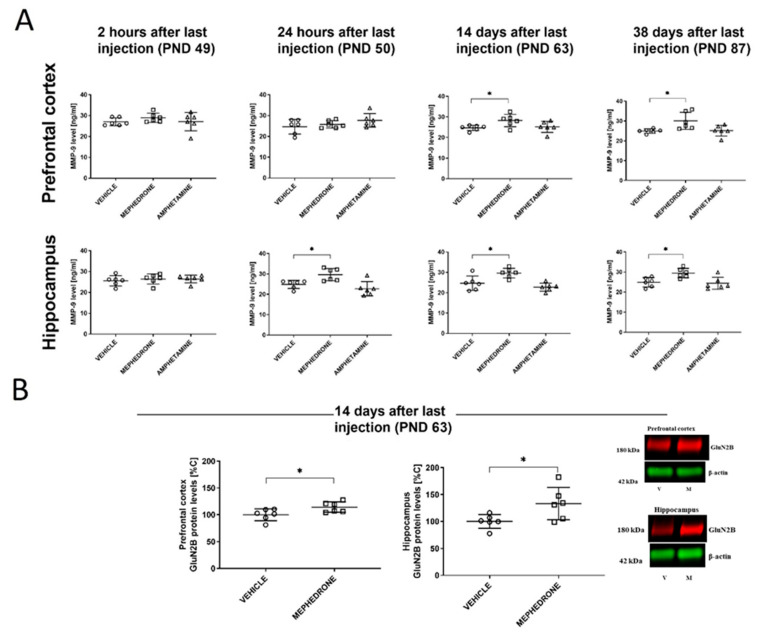
Effect of repeated mephedrone or amphetamine administration during adolescence on MMP-9 expression in the PC and HC of rats that did not undergo the Barnes maze task at different time (2 h, 24 h, 14, and 38 days) after the last administration (**A**). Effect of repeated mephedrone administration during adolescence on GluN2B subunit expression after 14 days of drug withdrawal (**B**). Representative blots for significant changes are presented, as well as full membranes are presented in the supplementary material (Figure S2). Data are expressed as the mean ± SEM. *n* = 6 rats/group. * *p* < 0.05 vs. vehicle (0.9% NaCl). V—vehicle, M—mephedrone.

Mephedrone (10 × 30 mg/kg, i.p.), but not amphetamine (10 × 2.5 mg/kg, i.p.), given repeatedly to adolescent rats had influence upon the expression of MMP-9 in the PC. However, one-way ANOVA did not reveal significant changes two hours (F(2,15) = 0.7809; *p* > 0.05) and 24 h after the last drug administration (F(2,15) = 1.647; *p* > 0.05). In contrast, a significant increase in MMP-9 expression was found after 14 days (F(2,15) = 3.754; *p* < 0.05; *n* = 6) and 38 days (F(2,15) = 5.611; *p* < 0.05). Dunnett’s post-hoc test revealed that mephedrone, but not amphetamine increased the MMP-9 expression level (*p* < 0.05) in the PC 14 and 38 days after the last administration of this drug (Figure 5A).

Similarly, mephedrone (10 x 30 mg/kg, i.p.), but not amphetamine (10 × 2.5 mg/kg, i.p.), given repeatedly to adolescent rats had influence upon the expression of MMP-9 in the HC. Here, one-way ANOVA did not reveal significant changes after 2 h (F(2,15) = 0.2798; *p* > 0.05) after the last drug administration. However, after 24 h (F(2,15) = 8.891; *p* < 0.01); 14 days (F(2,15) = 10.43; *p* < 0.01; *n* = 6); and 38 days (F(2,15) = 6.956; *p* < 0.01) of drug withdrawal, significant changes were noted. Dunnett’s post-hoc test also indicated that mephedrone increased MMP-9 level (*p* < 0.05) in the HC 24 h after the last administration. Furthermore, an increase of MMP-9 in this brain structure was observed 14 and 38 days (*p* < 0.05) after the last mephedrone administration (Figure 5A). Mephedrone (10 × 30 mg/kg), given repeatedly to adolescent rats had influence upon the expression of the Glun2B subunit of the NMDA receptor, Student’s t-test revealed significant changes in both brain structures. Thus, a significant increase of this subunit was observed in PC (t = 2.388; *p* < 0.05) and HC (t = 2.482; *p* < 0.05) (Figure 5B).

## 4. Discussion

The present finding shows that spatial learning deficits appear during adulthood in rats exposed to mephedrone or amphetamine during late adolescence. In addition, mephedrone but not amphetamine administration induced changes in MMP-9 level in the PC and HC—two regions crucial to the formation and recollection of long-term spatial memory. In adult rats (PND87) with memory deficits, significant changes were observed in the NMDA receptor subunits expression (GluN2B and GluN2A), as well as in the expression of PSD-95 in the PC and HC. Furthermore, changes in GluN2B subunit expression were found in the HC and PC in rats that did not undergo the learning task but were treated with mephedrone during adolescence. Although the differences in outcomes between groups were small in size, the groups of animals were homogenous and the number of animals per group achieved an optimal sample size to reach statistical significance; similar to the work of other authors that had assessed MMP-9 activity after administration of mephedrone [42]. Taken together, these findings suggest that recreational use of mephedrone/amphetamine by teenagers may induce cognitive dysfunctions in spatial memory that are seen in adulthood.

In Experiment 1, we evaluated the influence of repeated doses of mephedrone or amphetamine given during late adolescence on the spatial memory in adult rats. Previous study [40] revealed that mephedrone-treated rats during the peri-adolescent period (25 mg/kg, subcutaneously (s.c.), three times a day for two consecutive days) displayed an impairment of the reference memory in the Morris Water Maze (MWM) one week beyond the cessation of drug exposure, while the spatial learning process seemed to be preserved. Another study [37] showed that adult mice treated with mephedrone (30 mg/kg, twice daily for 4 consecutive days and tested 2–8 weeks following the final treatment) reduced working memory performance in the T-maze spontaneous alternation task but did not show deficits in the MWM performance. A further study demonstrated that mice given only one day mephedrone administration (four times at the dose of 25 mg/kg, s.c.) performed more poorly than the control in the MWM task one week after the cessation of drug exposure [39]. In turn, our study (Experiment 1) showed that adult rats that were repeatedly exposed to mephedrone during late adolescence (PND 40–49) displayed significant deficits in acquisition of spatial memory and showed increase in the primary latency and number of errors during the probe trial in the Barnes-maze task. These deficits were observed in the withdrawal period at three weeks (Probe trial 1: PND 71) and five weeks (Probe trial 2; PND 84). Thus, since these memory deficits were long-lasting, the effect is more likely to be a result of neurocognitive dysfunctions, including drug-induced long-term neuronal changes, rather than being due to a drug psychostimulant effect—as the animals did not show any changes in locomotion on the test day (Probe trial 1). The adult rats that received amphetamine during adolescence do not indicate a deficit in spatial learning in Probe trial 1 (indicating only tendency to memory impairment in the primary latency and number of errors) but indicated significant deficit in Probe trial 2 (PND 84). Such data are in accordance with that previously published in the sense that repeated amphetamine/methamphetamine administration during adolescence produces delayed, long-lasting deficits in learning and memory in adulthood [69,70,71,72]. However, we show for the first time that mephedrone, in contrast to amphetamine, induced a much stronger deleterious effect upon memory when given during adolescence. These deficits in memory performance were seen earlier (during acquisition training) and persisted into late adulthood.

Reversal learning is representative of flexibility and adaptability to a changing environment [73]. Published data revealed that mice that were treated eight weeks previous with mephedrone at the dose of 30 mg/kg, twice daily for four consecutive days, showed improvement in memory during the reversal probe trial in the MWM. However, in this experiment, mephedrone was given to adult mice [37]. Thus, such outcome could be due either to the ability of the animals to learn the new location or because the drug-treated animals more quickly forgot most of what they previously learned. In our study, mephedrone was given to adolescent rats that were still undergoing brain-development processes. Our experiment performed five weeks (PND 85–87) after mephedrone/amphetamine withdrawal in the Barnes maze task showed that mephedrone—and to a lesser extent amphetamine-treated rats were not able to adjust their response when the position of the escape box was changed in the reversal learning test. Because these animals showed especially intense deficits in memory processes associated with learned information on the probe day (Probe trial 1 and/or 2), therefore the reversal learning deficits in adult rats following repeated mephedrone/amphetamine administration in adolescence may have been due to spatial learning rather than cognitive flexibility impairments. We should note that animals with reversal learning deficits usually show perpetuation of drug-seeking behavior and relapse [74,75,76,77].

It is known that MMP-9 activity in the brain tissues becomes elevated in hippocampal-dependent memory tasks [53,55,78,79,80], in addiction [58] or after excitotoxic exposure [81]. In our study (Experiment 2), MMP-9 levels increased after 2–5 weeks of mephedrone withdrawal in the PC and HC in animals that did not undergo the Barnes maze task. The HC showed to be the most sensitive part of the brain in the mephedrone-treated rats because an increase in MMP-9 levels was observed as early as 24 h after the last treatment. Similar increase in the MMP-9 levels was observed in the mephedrone treated animals that underwent the Barnes maze task (PND 87). Thus, it is possible that changes in MMP-9 levels are not due to animal behavior performance; but are rather the result of mephedrone-induced cognitive impairment. In the case of amphetamine-treated rats, at the applied dose, there were no changes in MMP-9 level in such brain structures. Thus, we can suggest that in these animals, similarly to methamphetamine-treated mice, MMP-9 is not a marker of neurodegeneration [82] but may only be involved in CNS remodeling.

MMP-9 is released from the postsynaptic compartment of excitatory synapses in an activity-dependent manner [83,84]. Upon activation, MMP-9, through cleavage of specific target proteins (an integrin ß1 -dependent pathway), regulates NMDA receptors mobility and function at the synapse, and appears to be a highly potent regulator of NMDA receptor surface trafficking [85,86,87]. The transient function of MMP-9 is required for maintenance of the late phase of NMDA-dependent LTP in various brain structures important for spatial memory, such as the HC [53,59] or PC [88]. However, the overexpression of MMP-9 leads to an increase of basal excitatory synaptic transmission and impairs synaptic plasticity [89]. In our study, mephedrone withdrawal induced a rapid increase in MMP-9 levels in HC and PC brain structures (24 h in HC and 14 days in PC) that appeared to impair cognitive processes in rats. The excessive MMP-9 activation may give rise to excitatory synapse morphological and functional changes in the PC or HC [90]. Notably, mephedrone and amphetamine produce unique, age-dependent effects on glutamate in these brain structures [81,91]. Therefore, we hypothesize that mephedrone exposure similar to amphetamine [92], influences glutamate release in the PC and HC and this effect, in the case of mephedrone co-exists with MMP-9 overexpression.

We noted up-regulation of the GluN2B subunit of NMDA receptor in the PC and HC in adult rats that received mephedrone in adolescence and had undergone the Barnes maze task. In adult rats that received amphetamine in adolescence, the GluN2A subunit in the HC (Experiment 1) was downregulated. Data has shown that PSD-95, a prototypical scaffolding protein present at excitatory synapses, is able to concentrate NMDA receptors at the synapse [93]. In our study, PSD-95 level was increased in the PC, but decreased in the HC. However, a limitation of our study is the using of the whole PC/HP homogenates instead of (crude) synaptosomal fractions. Nonetheless, published data indicate that GluN2B subunit expression in the synaptic membrane declines during aging [94,95]. Moreover, an increase association of GluN2B-containing NMDA receptor with PSD-95 in aged animals may have contributed to spatial memory decline [95].

Our current study demonstrates an increased PSD-95 and GluN2B expression in the PC of adult rats. Such outcome can suggest the presence of this receptor subunit (in the synaptic membrane) and decreased process memory. Hence, these data support our hypothesis that deficits observed in the Barnes maze task (Experiment 1) in the reversal learning (that is cortex-dependent process) may have been due to an impairment of spatial memory processes rather than cognitive flexibility impairment. In our hippocampal study, the GluN2B subunit expression was increased, but PSD-95 expression was decreased during the mephedrone withdrawal. Such data suggests that the GluN2B subunit is located mainly outside the synapses and might be a consequence of excessive glutamate release in this region. The observed effect seems to be related to the learning and memory impairment observed in Probe trials 1/2.

Our data showed that the GluN2B subunit of NMDA receptors that plays a crucial role in the early stages of brain development [91,94,95], is specifically affected by mephedrone administration. The upregulation of this subunit was observed in brain slices of animals that did not undergo and did undergo the memory tasks. Moreover, in both groups of animals, MMP-9 levels were increased. Thus, we can hypothesize that there is causal link between these two parameters.

In our study, amphetamine withdrawal reduced the PSD-95 level and decreased GluN2A subunit expression in the adult rats HC without an influence on GluN2B subunit. Thus, our result suggests a lowered expression of the GluN2A subunit in hippocampal synapses. The decline in GluN2A subunit in adult rats may infer a decreased efficacy of synaptic transmission during synaptic plasticity processes. Published data suggests that the GluN2A subunit of NMDA receptor is not necessary for long-term memory tasks but seems to affect short-term memory and the rapid acquisition of spatial information [96]. Although GluN2B subunit also affects short-term memory, it may make a greater contribution to learning when information must be retained after a longer delay or there is incremental task acquisition across a number of days [93]. Taking into account that GluN2A expression increases with age [97,98], while GluN2B expression is high at birth, but decreases in adulthood, the above data suggest that mephedrone administration during adolescence can induce more harmful effects than amphetamine on the adolescent brain.

A few limitations of the current study are worth noting. First, we used rats to model human drug addiction. Although these are the preferred animal for such type of study, they do not fully mimic human drug addiction behavior [99]. We also analyzed whole brain structures and whole PC/HP homogenates instead of (crude) synaptosomal fractions. Moreover, to support our hypothesis on the relationship between NMDA receptors (especially GluN2B) and MMP-9, in mephedrone-induced memory deficits we should have used genetically modified animals with altered NMDA receptors. Future studies are, therefore, needed to control for the potential influence of the aforementioned factors in the mephedrone/amphetamine-induced cognitive dysfunction observed in the present study.

In summary, our study indicates that repeated mephedrone, and to a lesser extent, amphetamine administration during late adolescence induces spatial memory deficits in adult rats. Furthermore, cognitive flexibility impairments result from spatial learning impairments. In addition, mephedrone but not amphetamine administration enhanced, with delayed onset, MMP-9 level in the PC and the HC of rats that did not undergo testing. In contrast, in adult rats that underwent the Barnes maze task, the mephedrone-induced memory deficits are paralleled by increased MMP-9 levels in the PC and HC, and (as we show for the first time) alterations in the NMDA receptor subunits and PSD-95 expression.

Taking into account our results, we conclude that mephedrone used during adolescence has a deleterious effect on cognitive processes in adulthood and this phenomenon could be associated with MMP-9 over-expression and GluN2B subunit up-regulation in such brain regions as the PC and HC.

## Figures and Tables

**Table 1:** Effect of repeated mephedrone or amphetamine administration on locomotor activity.

Effect of Repeated Mephedrone or Amphetamine Administration on Locomotor Activity Measured Probe Trial-1 Day (PND 71) of Barnes Maze Task		
Compounds:	N	Distance traveled (m) ± SEM
Vehicle	8	56.24 ± 1.972 (NS)
Mephedrone (3 × 10 mg/kg)	8	66.16 ± 3.574 (NS)
Amphetamine (3 × 2.5 mg/kg)	8	60.22 ± 3.840 (NS)

## Data Availability

The data presented in this study are available on request from the corresponding author.

## Supplementary Materials

The following are available online at https://www.mdpi.com/1422-0067/22/2/589/s1. Figure S1. Corresponding membranes from Western blot analyses of NMDA receptor subunits and loading controls in the prefrontal cortex (A) and hippocampus (B). Figure S2. Corresponding membranes from Western blot analyses of NMDA receptor subunits and loading controls (β-actin) in the prefrontal cortex (A) and hippocampus (B).
