# Supplementary material for: Effects of Mephedrone and Amphetamine Exposure during Adolescence on Spatial Memory in Adulthood: Behavioral and Neurochemical Analysis
Source: Int J Mol Sci. 2021 Jan 8;22(2):589. doi: 10.3390/ijms22020589 (PMC7827725; doi:10.3390/ijms22020589)
Supplement: Supplementary file 1 [file ijms-22-00589-s001.pdf]

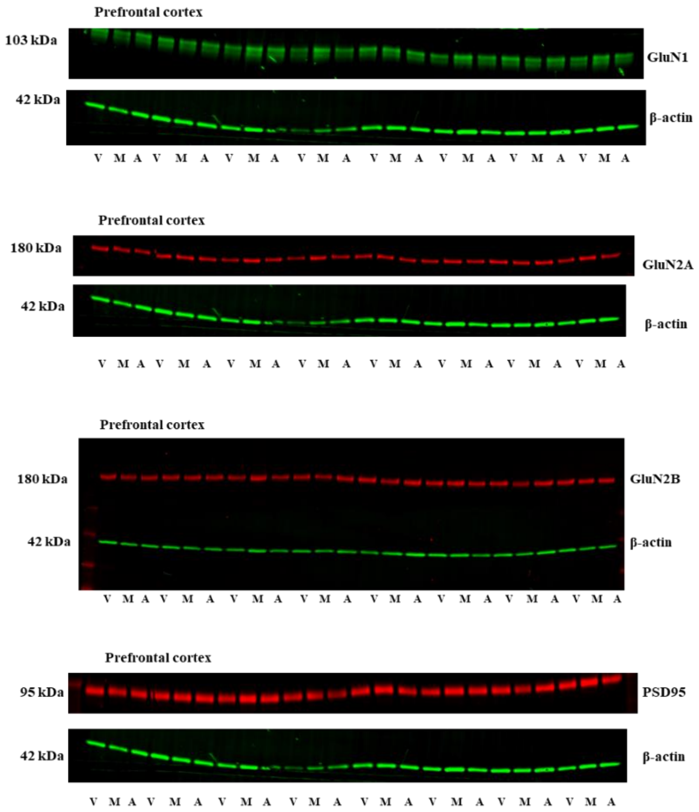

(A)

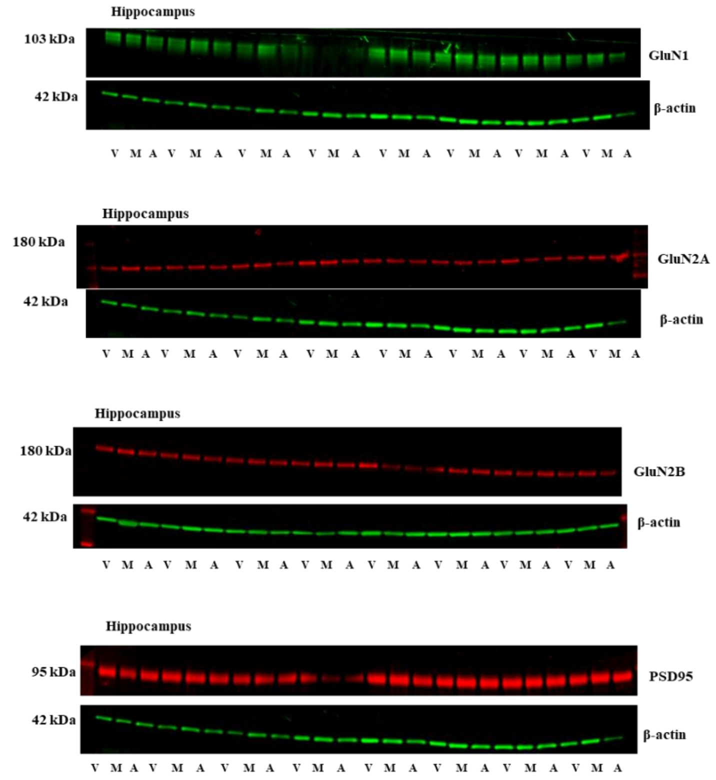

(B)

Figure S1. Corresponding membranes from Western blot analyses of NMDA receptor subunits and loading controls in the prefrontal cortex (A) and hippocampus (B). V- vehicle, M- mephedrone, A- amphetamine. Control protein (β-actin) for GluN1, GluN2A, and PSD-95 was the same.

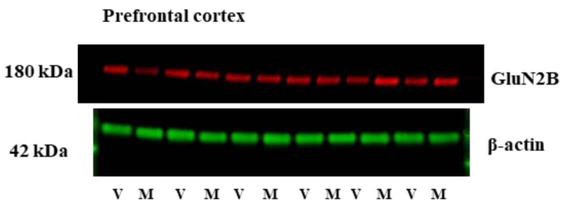

(A)

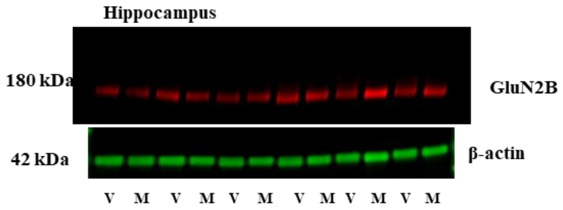

(B)

Figure S2. Corresponding membranes from Western blot analyses of NMDA receptor subunits and loading controls (β-actin)in the prefrontal cortex **(A)** and hippocampus **(B).** V- vehicle, M- mephedrone.
